# Overtube-assisted technique for insertion of linear endoscopic ultrasonography

**DOI:** 10.1093/gastro/goz015

**Published:** 2019-05-25

**Authors:** Xin-Xing Tantai, Mo Wang, Xiao-Dan Guo, Jin-Hai Wang, Shi-Yang Ma

**Affiliations:** Division of Gastroenterology, The Second Affiliated Hospital, Xi'an Jiaotong University, Xi'an, Shaanxi, P. R. China

## Introduction

Over the last three decades, endoscopic ultrasonography (EUS) and EUS-guided fine-needle aspiration (EUS-FNA) have gradually become established tools for the diagnosis, staging and treatment of benign or malignant gastrointestinal diseases and pulmonary disorders [1]. Compared with standard upper endoscope, EUS uses a dedicated echoendoscope that has a more rigid tip and a longer nonflexible segment at the most distal end of the device. On the other hand, most currently available linear echoendoscopes are side-viewing instruments. Both the unique mechanical and optical features of EUS scopes make manipulations difficult; even skilled EUS operators occasionally encounter difficulty when inserting, which may be more likely to cause severe complications [2]. One of the most frequent complications is gastrointestinal perforation, which can take place in the hypopharynx, hiatal hernia, tip of the duodenal bulb and rectosigmoidal junction [3]. The incidence rate of pharyngoesophageal perforations resulting from gastrointestinal endoscopes is approximately 0.01% [2]. For EUS, a higher risk of perforation has been reported [4]. Hypopharyngeal perforation can lead to lethal complications (e.g. mediastinitis, mediastinal pneumothorax and fistulas) and these complications are associated with a mortality rate of 2%–36% [5]. Therefore, it is necessary to explore some techniques to avoid these complications to the utmost extent.

An endoscopic overtube is used as a safety device or a facilitator in gastrointestinal endoscopy, which can protect the hypopharynx and esophagus of patients who need multiple esophageal intubations or retrieval of foreign bodies. We report here the novel use of such an endoscopic overtube to facilitate the insertion of linear EUS.

### Overtube-assistant technique

#### EUS instruments

The EUS procedure was performed when the patient was in the left lateral recumbent position and, if necessary, conscious sedation was provided using midazolam (10–15 mg iv.). All procedures were performed using Pentax 3870UTK Linear and Pentax 3270UK linear endoscopes with Hitachi AVIAS sonography and common gastroscopic examination used a Pentax EG29-i10 with i-7000. The overtube (MD-48719) was provided by Sumitomo Corporation. The inner dimension of the overtube was 16 mm and the outer dimension 19 mm, with a length of 20.5 cm.

### Standard intubation procedure

The distal end of the EUS scope was pressed close to lingual surface and pushed cautiously, with the tip of the ultrasound probe point to the left of the piriform recess or the middle of the posterior pharyngeal wall after passing the palatopharyngeal arch, the resistance decreasing when asking the patient to swallow, then pushed slightly and inserted into the esophagus. If the resistance from the distal end was high or the scope was unable to move forward, the location of the ultrasound probe should be adjusted slightly or tried to insert into the esophagus from the right piriform recess if necessary. When the above adjustments would not help, the result was recorded as a failure. In the second insertion, the operator could make another similar standard attempt or choose the overtube-assisted technique. However, the attempt at intubation should not be made more than three times.

### Overtube-assisted procedure

**Figure 1.**
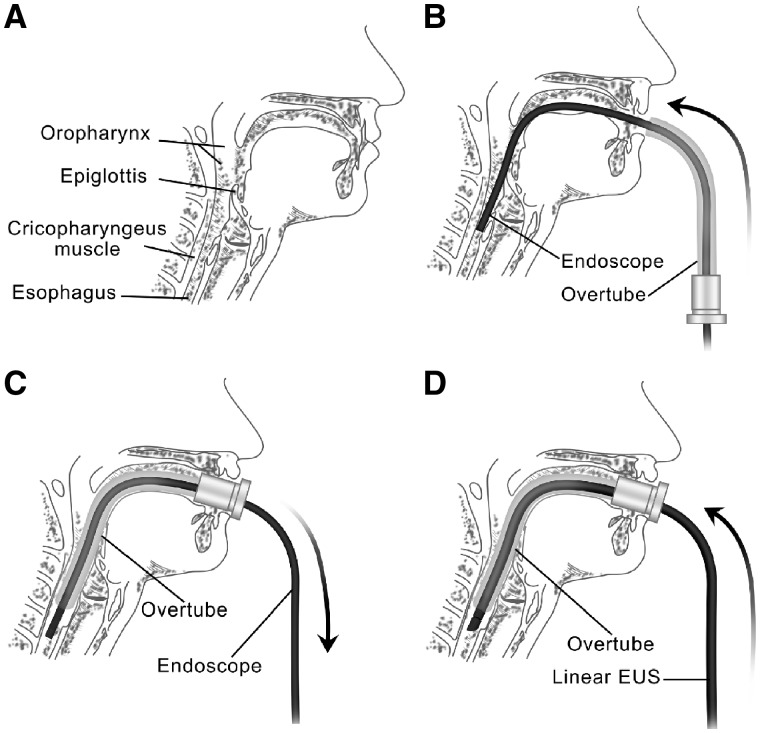
Overtube-assisted endoscopic ultrasonography (EUS). (**A**) Relevant anatomic landmarks of the upper digestive tract. (**B**) The overtube was pushed into the esophagus along the endoscopic body after the gastroscope was intubated successfully. (**C**) The gastroscope was pulled out and the overtube was left for the insertion of the EUS scope. (**D**) The EUS scope was inserted through the retained overtube.

If the standard insertion procedure of the oropharynx could not be completed, we would adopt the overtube-assisted technique immediately (Figure 1). The normal gastroscope with an overtube fastened outside close to the handle would be intubated routinely through the oropharynx. The damage to the pharynx would be imaged and evaluated before insertion of the scope into the esophagus. Then a lubricated overtube would be inserted into the oropharynx along the scope when the scope reached the middle segment of the esophagus. Then the scope would be pulled out, while the overtube was left for insertion of the EUS scope. Finally, the linear EUS scope would be inserted along the overtube, enabling subsequent operations. After the EUS procedures, the conditions of the pharynx would be evaluated for the second time under direct visualization with the normal endoscope. All the above operations were performed step by step and in the same session.

### Practical application

**Figure 2.**
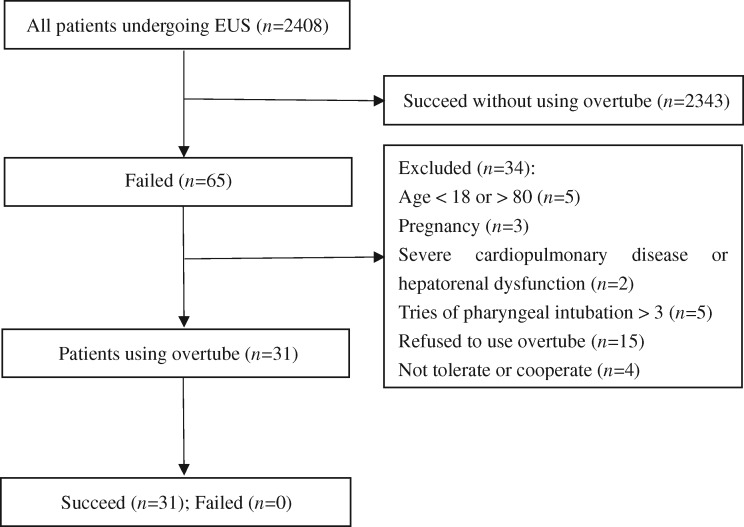
Flow chart of patients who underwent endoscopic ultrasonography (EUS) using the overtube-assisted technique

Between March 2016 and February 2018, 31 outpatients who underwent overtube-assisted EUS at the Endoscopy Center, The Second Affiliated Hospital of Xi’an Jiaotong University (Shaanxi, China) were included (Figure 2). There were 14 males and 17 females with a mean age of 57.5 years (range, 23–80 years). Nine patients were given intravenous injection of midazolam for conscious sedation. In order to describe the procedure-related complications, all patients were evaluated twice by gastroscopy. The first evaluation was to identify the condition of the pharyngeal injury due to the standard intubation procedure and the second assessment was to determine whether using the overtube aggravated mucosal injury.

Of the 31 patients, 18 underwent one try for pharyngeal intubation, among whom 4 presented with hyperemia edema or erosion and 14 showed normal mucosa. Nine patients underwent two attempts, among whom six developed erosion or hyperemia edema and three localized bleeding or hematoma. Four patients underwent three attempts and all showed serious injuries, among whom three developed localized bleeding and one localized hematoma.

Without repetitive operation, all patients completed pharyngeal intubation smoothly after using the overtube-assisted technique. In the second gastroscope evaluation, all mucosal conditions were not aggravated. No ulcers or perforations occurred after all the procedures. Patients were discharged home from our recovery area after 2 hours of post-procedural monitoring. A week later, patients were asked for a telephone interview and all the patients had no pharyngeal discomfort.

## Discussion

EUS has been widely applied in clinical practice for decades and its indications can be divided into several categories: evaluation of gastrointestinal malignancies, submucosal abnormalities, pancreatic-biliary diseases, mediastinal diseases, evaluation of perianal diseases, extraluminal abnormalities identified on other imaging examination and relevant therapeutic applications [1]. However, the manipulation of the EUS scope is difficult and it easily perforates the gastrointestinal wall, particularly in areas of angulation (e.g. the hypopharynx) [3].

For difficult hypopharyngeal intubation of the oblique-viewing endoscope, previous studies had tried some creative techniques. Malik *et al*. [6] described a technique using a hydrophilic guide wire. However, patients had to cooperate by swallowing either the catheter or the guide wire first before insertion of the duodenoscope. Tsang *et al*. [7] described the use of catheter guidance in difficult cases. A catheter was advanced through the channel of the endoscope into the esophageal inlet under direct vision, then intubation of the esophageal inlet was accomplished over the catheter. Wai *et al*. [8] described a case of difficult intubation during an endoscopic retrograde cholangiopancreatography (ERCP). By using a forward-viewing gastroscope, a guide wire was inserted via the working channel into the stomach and then the duodenoscope with a cannulating catheter was advanced into the esophagus over the guide wire. However, these methods may be time-consuming and unsuitable for the EUS scope, which is thicker than a duodenoscope.

In the current study, 31 patients completed pharyngeal intubation smoothly using the overtube-assisted technique and pharyngeal mucosa conditions were not aggravated based on the second gastroscopy evaluation. Before applying the overtube-assistant technique, two patients in our center underwent pharyngeal perforation after three and five standard attempts, respectively, which were finally confirmed to be caused by pharyngeal stenosis (swollen thyroid gland and lung cancer with its metastasis in the mediastinum, respectively). Although this cohort of patients did not include cases with esophageal stenosis, other researchers have proved that the overtube-assisted technique may be effective in patients with esophageal stenosis [9–11].

It is understandable that the number of tries of the initial pharyngeal intubation is proportional to the risk and severity of the mucosal injury. All patients who had intubation attempted three times developed serious mucosal injuries. Therefore, we think it is appropriate that the number of tries of the initial pharyngeal intubation should not be beyond two. For beginners who practice EUS, timely use of the overtube will help to reduce the incidence of serious complications. Previous research has shown that older patient age (≥65 years), lack of operator experience, a history of difficult intubation and the presence of a cervical pathologic condition may be risk factors for cervical perforation during upper EUS examination [4]. For patients with the above risk factors, the overtube-assisted technique may be beneficial.

In conclusion, the overtube-assisted technique may be a safe and effective method for pharyngeal intubation of the linear EUS scope. In case of difficult esophageal intubations, using an overtube may be an alternative method.

## Authors’ contributions

X.X.T.: writing of the manuscript and study conception and design; S.Y.M.: contribution to writing and revising the manuscript, reading images, study conception and design, and performing EUS; M.W.: data collection and reading images; J.H.W. and X.D.G.: reading images.

## Funding

This work was supported by the grant from the Shaanxi Provincial Key Research and Development Program [No. 2018SF-191 to S.Y.M.].
